# 

**Published:** 1783

**Authors:** 


### PROMOTED.

M. de Laſſone, firſt phyſician to the queen of France, Dr. John Rogerſon, firſt phyſician to the empreſs of Ruſſia, and Dr. John George Zimmermann, firſt phyſician to his majeſty at Hanover, to be honorary fellows of the royal college of phyſicians at Edinburgh.

1783, *March* 29. Mr. John Groves to be ſurgeon to the 30th regiment of foot.

*April* 19. Mr. John Teare to be ſurgeon to the 85th regiment of foot.

*May* 2. W. H. Muckleſton, M. B. late phyſician to the infirmary at Shrewſbury, to be phyſician to St. George's hoſpital in London, in the room of Dr. Matthews, who has reſigned.—20. Mr. William Fleming, ſurgeon's mate of the 56th, to be ſurgeon to the 25th regiment of foot.—Mr. Robert Morris, hoſpital mate, to be ſurgeon to the 59th regiment of foot.—24. Mr. James Snagg, ſurgeon's mate, to be ſurgeon to the 14th regiment of foot.—Mr. Thomas Harriſon to be ſurgeon to the 18th regiment of dragoons.—27. Mr. Charles Hall, late ſurgeon to the 14th regiment of foot, to be ſurgeon to the hoſpital in Jamaica.—Mr. Everard Home, aſſiſtant ſurgeon, to be apothecary and ſtorekeeper to the hoſpital in Jamaica.—Mr. John Lorimer to be ininſpector of regimental hoſpitals in North America.

*June* 21. Mr. John Foot, ſurgeon of the late 113th regiment, to be ſurgeon to the forces in Canada.—John Watkinſon, M. D. to be phyſician to St. Thomas's Hoſpital in the room of the late Dr. Keir.

---

### D I E D.

1781, *Jan.* Chr. Philip Herwig, M. D. firſt phyſician to the prince of Hohenloh Waldenburg.

1782, *Jan.* 21. At Frankfort on the Mayne, aged 39 years, J. J. Reichard, M. D. profeſſor of Botany.

1783, *Jan.* At Kingſton in Jamaica, aged 33 years, Mr. John Pearce, navy-ſurgeon, and member of the corporation of ſurgeons and ſociety of apothecaries in London.—At Calais, M. Froiſſart, M. D.

*March* 12. At Liſbon, of a pulmonary conſumption, aged 32 years, Patrick Dugud Leſlie, M. D. F.R.S. late phyſician at Durham, author of a very ingenious diſſertation *de Caloris Animalium Cauſa*, printed at Edinburgh in 1775, and afterwards greatly enlarged and publiſhed in Engliſh, under the title of "A Philoſophical "Enquiry into the cauſes of animal heat, 8vo. "London, 1779."—29. In Berner's Street, London, Benj. Cowell, eſq. formerly ſurgeon to St. Thomas's Hoſpital.—30. At his houſe in Great

Windmill Street, London, in his 67th year, Dr. William Hunter, F.R.S. & S.A. member of the college of phyſicians, &c. and phyſician extraordinary to the Queen. We hope ſoon to be enabled to preſent our readers with ſome authentic memoirs of this deſervedly celebrated phyſician.

*April.* In Stafford Street, Dublin, Daniel Rainey, M.D. author of an inaugural theſis *de Peripneumonia vera*, printed at Leyden in 1764.—13. At Newark upon Trent, William Stevenſon, M.D. author of an inaugural diſſertation *de Diabete*, printed at Edinburgh in 1762; and of "A method of treating the gout by bliſtering, "8vo. London 1779;" a hiſtory of the American War in 4 vols. 8vo. and other works. (See p. 182. of vol. III. and p. 104 of our preſent volume)—20. In Grafton Street, Dublin, in his 29th year, William Cleghorn, M.D. nephew of the celebrated Dr. George Cleghorn, who reſigned in his favour the office of lecturer on anatomy in Trinity College, Dublin. This ingenious young phyſician graduated at Edinburgh in 1779, on which occaſion he defended a theſis, *de Igne*.

*May* 14. At Montpellier, advanced in years, M. Mejan, a very ingenious ſurgeon, and author of a method of treating the Fiſtula Lachrymalis, deſcribed in the 2d volume of the *Mem. de l'Acad. de Chirurgie*.—19. At Chewbent in Lancaſhire, Mr. William Bagſhaw, ſurgeon and apothecary.—22. Near Briſtol, Mr. William Wray, formerly an apothecary in Covent Garden.—23. In Golden Square, Mr. John Norton, member of of the corporation of surgeons of London.— 25. In Basinghall Street, London, Mr. Joseph Adams, one of the court of assistants of the company of apothecaries in London.—30. At Whitchurch in Shropshire, Mr. John Wickstead, surgeon and apothecary.

*June* 4. At Biggleswade, on his return from London, Henry Smith, M.D. physician at Doncaster in Yorkshire, and author of an inaugural dissertation *de Magnesia Alba*, printed at Edinburgh in 1752.—6. At Canterbury, to which place he retired about a year ago, Thomas Lawrence, M. D. in the university of Oxford, and formerly president of the royal college of physicians in London, of which he was elected a fellow in the year 1744. He was a man of great integrity and erudition, and of considerable anatomical knowledge, much of which he acquired under the tuition of Dr. Frank Nichols, to whose memory he has paid a very elegant tribute in Latin. His dissertation *de Hydrope*, and his other works, are deservedly admired for the classical style in which they are written, but, like those of his master Dr. Nichols, are too much tinctured with the principles of the Stahlians.——Same day in Hatton Street, London, aged 30 years, William Keir, M.D. member of the college of physicians, physician to St. Thomas's Hospital, and lecturer on chemistry in London; and member of the medical and philosophical societies at Edinburgh. This worthy and ingenious physician, whose premature death is much and deservedly lamented, was a native of the county of Perth in North Britain, and nenephew of Dr. Orme, phyſician in London. He graduated at Edinburgh in 1778, on which occaſion he defended a theſis *de attractione chemica*. In 1780 he was elected phyſician to St. Thomas's Hoſpital in London, and the ſame year married a daughter of Mr. James Rae, ſurgeon in Edinburgh, by whom he has left iſſue two children. —9. At Liverpool, Mr. William Preſton, ſurgeon.——12. At Briſtol Wells, Mr. Atkinſon, lately an apothecary at Chelſea.—15. At Lambeth, advanced in years, Mr. John Naſh, late ſurgeon and apothecary at Sevenoaks in Kent.—17. At Barnſley, in Yorkſhire, Mr. Robert Dymond, ſurgeon and apothecary in Holborn, London.

---

### SECTION IV.

### Quarterly Catalogue.

1. SOME account of the late John Fothergill, M.D. Member of the Royal College of Phyſicians, and Fellow of the Royal Society of London, Fellow of the Royal College of Phyſicians in Edinburgh, and Correſponding Member of the Royal Medical Society of Paris, and of the American Philoſophical Society at Philadelphia. By *John Coakley Lettſom*. 8vo. *Dilly*, London, 1783. 193 pages.

This

This volume contains a great many intereſting anecdotes of which we have availed ourſelves in our account of Dr. Fothergill (page 176). It is intended to bind up with an edition of his works, of which we mean to give an account as ſoon as it is completed.

2. Chirurgical Eſſays on the cauſes and ſymptoms of ruptures; their natural conſequences if neglected; and the danger of the truſſes applied by workmen; with caſes to illuſtrate an improved method of treatment and cure. By *T. Brand*, Member of the Corporation of Surgeons, London, and Surgeon Extraordinary to his Majeſty's Royal Hoſpital at Greenwich. 8vo. *Dodſley*, London, 1783. 2 s.

3. Remarks on Mr. Brand's Eſſays on the cauſes and ſymptoms of ruptures, &c. with a ſhort but true hiſtory of the invention of Mr. Brand's patent elaſtic truſſes. By *T. Sheldrake*, junior. 8vo. *Stockdale*, London, 1783.

4. Obſervations on the management of diſeaſes of the army and navy, during the American war; together with ſome account of the loſs of Senegal, and of the army at York in Virginia; in reply to Dr. Monro. By *John Millar*, M. D. 4to. *Stockdale*, London, 1783. 60 pages.

5. An

5. An Eſſay on the ſymptoms and cure of the virulent Gonorrhœa in females. By *C. Armſtrong*, Member of the Corporation of Surgeons of London, and Accoucheur. 8vo. *Dilly*, London, 1783. 1 s.

6. *Collectanea Hibernica Medica*, N° I. Being a collection of and repoſitory for papers of advice, diſcuſſion and reſearch in all departments of medicine, intended not only to hand to poſterity, in abſtract pieces of diſquiſition, the reſult of our improvements in that line, but alſo to excite a ſpirit of phyſical and medico-phyſical inquiry through this kingdom. By *Richard Harris*, M. D. phyſician at Clonmel. 8vo. Dublin, 1783. 2 s. 2 d.

7. Caſes in Midwifery; with references, quotations, and remarks. By *William Perfect*, ſurgeon, of Weſt Malling, in Kent. Vol. II. 8vo. *Fiſher*, Rocheſter, 1783. 6 s.

8. Reports of the Humane Society, inſtituted in the year 1774, for the recovery of perſons apparently drowned. For the years 1781 and 1782. 8vo. *Rivington*, London, 1783. 162 pages.

Dr. Hawes, the editor of this publication, intends, we are informed, to compile from theſe and the preceding reports of the Humane Society, ciety, the moſt intereſting caſes of reſtoration that have occurred ſince its inſtitution. Such a collection will certainly be very acceptable to the medical reader.

9. Memoirs of Albert de Haller, M. D. Member of the Sovereign Council of Berne, Preſident of the Univerſity and of the Royal Society of Gottingen, Fellow of the Royal Society of London, &c. Compiled, chiefly, from the eulogium ſpoken before the royal academy of ſciences at Paris, and from the tributes paid to his memory by other foreign ſocieties. By *Thomas Henry*, F. R. S. Member of the Medical Society of London, and of the Literary and Philoſophical Society of Mancheſter. 12mo. *Johnſon*, London, 1783. 2 s. 6 d. ſewed.

We have peruſed this little volume with great pleaſure. The materials are drawn from authentic ſources, and are judiciouſly arranged. In a poſtſcript Mr. Henry has given a liſt of Haller's correſpondents in this country, and prefixed to the work is an engraved portrait, which, from our perſonal knowledge of M. de Haller, we can venture to recommend as a ſtriking likeneſs of that truly great man.

10. An Analyſis of the ſection of the ſymphyſis of the oſſa pubis, as recommended in caſes of of difficult labour and deformed pelvis, by Dr. Alphonſo le Roy, profeſſor of midwifery at Paris. By *James Rymer*, ſurgeon. 8vo. *Evans*, London, 1783. 27 pages. 1 s.

11. An Inquiry, by experiments, into the properties and effects of the mineral waters in the county of Eſſex. By *W. Martin Trinder*, L. L. B. at Oxford, and M. D. at the univerſity of Leyden. 8vo. *Rivington*, London, 1783. 36 pages. 1 s. 6 d.

12. Verſuch einer geſchichte des mineral reichs. *i. e.* An Eſſay towards an hiſtory of the mineral kingdom. By *C. A. Gerhard*. Vol. I. 8vo. Berlin, 1782. 342 pages.

13. Diſſertation Anatomico-acouſtique. *i. e.* An Anatomico-acouſtic Diſſertation. By *M. Perrolle*, M. D. Correſponding Member of the Royal Academy of Sciences at Montpellier. 8vo. Paris, 1783. 42 pages.

This work is divided into two parts. In the firſt the author endeavours to prove, that ſounds do not enter the Euſtachian tube. In the ſecond he gives an account of experiments made at Paris in 1777 by Abbé de Lépee with the Deaf and Dumb.

14. Aſſembleé publique de la Société Royale des Sciences tenüe dans la grande ſalle de l'hotel de de cette ſociété, en preſence des etats de la province de Languedoc, le 27 Decembre, 1781; *i. e.* An account of the public meeting of the Royal Society of Sciences, held in the hall belonging to the ſociety, in the preſence of the ſtates of the province of Languedoc, December 27, 1781. 4to. Montpellier, 1782.

This collection contains the eulogy of M. René Gaſpard de Joubert; a memoir on the manufacture of Flint Glaſs, by M. Allut; experiments on the uſe which may be derived from the vinous gas, by M. Mourgue; new proofs of the efficacy of conductors (*Paratonnerres*) by Abbé Bertholon; and remarks on the Tænia, by M. Cuſſon, junior, M. D.

15. Recueil des Memoires et d'Obſervations, tant ſur les maladies qui attaquent l'œil et les parties qui l'environnent, que ſur les moyens de les guerir, dans lequel l'auteur, après avoir donné un precis de la ſtructure de cet organe, expoſe un nouveau procédé pour extraire la cataracte avec un inſtrument de ſon invention, & réfute l'efficacité pretendüe de l'abbaiſſement; *i. e.* A collection of Eſſays and Obſervations, as well on the diſeaſes which attack the eye, and the parts that ſurround it, as on the means of curing them, in which the author, after giving an account of the ſtructure of that organ, points out a new proceſs for the extraction of the cataract, with an inſtrument of his invention, and refutes the pretended efficacity of depreſſion. By M. *G. Pellier de Quengsy*, fils. 8vo. Montpellier, 1783. 549 pages.

16. *C. Gaſpar Siebold*, M. D. & Prof. &c. Parotidis Schirroſæ feliciter extirpatæ hiſtoria. 4to. Erfurti, 1781. p. 12.

17. De Analyſi Urinæ et acido Phoſphoreo Commentarius. Auctore *Thoma Lauth*. 4to. Straſburgh, 1780. p. 54.

18. *Bernardi Nicolai Pluvinet*, Pariſini, Tentamen Chemicum de Fermentatione Spirituoſa et Acetoſa, harumque productis. 4to. Monſpeliæ, 1781. p. 42.

19. Lezioni intorno alle Malattie degli occhi, &c. *i. e.* Lectures on the diſeaſes of the eyes, for the uſe of the new college founded by the King of Naples at the Hoſpital for Incurables. By *Michael Troja*, Regius Profeſſor in that college. 8vo. Naples, 1731. 403 pages, with two copper plates.

20. *Henr. Aug. Wriſbergii* Profeſſoris Gottingenſis Experimenta et Obſervationes Anatomicæ de Utero Gravido, tubis ovariis et corpore luteo quoquorumdam animantium cum iisdem in homine collatis. 4to. Gottingæ, 1782. p. 40.

21. Journal des Observations Mineralogiques faites dans une partie des Vosges, et de l'Alsace, ouvrage qui a remporte le prix au jugement de M. M. de la Société Royale des Sciences, Belles Lettres, et Arts de Nancy en 1782. *i. e.* A Journal of Mineralogical Observations made in a part of the Mountains of Vosges, and of Alsace, being the work which obtained the prize from the Society of Sciences, Belles Lettres, and Arts at Nancy in 1782. By *M. Sivry*, advocate, 8vo. Nancy, 1782. 117 pages.

22. Physikalisch Chemische Untersuchung der thienschen feuchtigkeiten. *i. e.* A Physico-Chemical Inquiry concerning the animal fluids. By *J. A. Weber*, counsellor of state to the comte de Weid, and member of the society of sciences at Rotterdam. 8vo. Tubinguen, 1781. 140 pages.

23. Abhandlung von dem Salze. *i. e.* A Treatise on Salt. By *Lewis Rousseau*, M. D. professor of chymistry, natural history, and materia medica in the university of Ingolstadt. 8vo. Ingolstadt 1781. 198 pages.

24. De Antiqua Medico-philosophia orbi novo adaptanda, oratio habita in Capitolio Gulielmolielmopolitano in Comitiis Univerſitatis Virginiæ, Die 12 Junii 1782, dum favente Gallorum Ducum et Militum frequentia, Medicæ Cooptationis Laureâ donabatur Chriſtianiſſimi Regis Exercitûs Archiater, *Joannes Franciſcus Coſte*, ſaluberrimarum Medicinæ Facultatum Pariſienſis alumnus, Valentinæ Doctor, Pennſylvanienſis Doctor honorarius; Regiarum Medicinæ Societatum Londinenſis, Edinburg, Paris. Regii Lotharingorum Medicorum Collegii honorarius; e Regiis Scientiarum Academiis Nanceiana, Lugdunenſi, Divionenſi; ex humanâ Societate Philadelphienſi; Societatis Philoſophicæ Americanæ Socius; Arcis et Militaris Noſocomii Caleti, Navaliumque Regis Exercituum Medicus. 8vo. Lugduni Batavorum, 1782. p. 103.

This academic diſcourſe, which is written with great elegance, is inſcribed to General Waſhington. The learned author, who officiated as firſt phyſician to the French army under M. de Rochambeau, is already advantageouſly known by ſeveral medical productions, particularly by a French tranſlation of Dr. Mead's works, in 2 vols. 8vo. with notes.

25. Diſſertatio Medica ſiſtens cauſas difficilis deglutitionis. Auctore *Carolo Chriſtiano Haaſe*. 4to. Gottingæ, 1781. p. 22.

26. Theorie

26. Theorie des Schielens. *i. e.* Theory of Strabismus. By *John Nepomucene Fischer*, Professor of Mathematics at Ingolstadt. 8vo. Ingolstadt, 1781. 71 pages.

27. Hippocratis Aphorismi ad fidem veterum monumentorum castigati, Latine versi, à *J. B. Lefebure*, M. D. 16mo. Paris 1782. p. 371.

28. *Caroli Godofredi Hagen* Tentamen Historiæ Lichenum. 8vo. Koningsberg, 1782. 182 pages, with coloured plates.

This work is divided into two parts. In the first the author treats of lichens in general, and in the second those which he himself has seen. He describes eighty species, five of which are new.

29. Dissertatio Inauguralis Medica de lichene Islandico, auctore *Gulielmo Christiano Phil. Cramer.* 4to. Erlangen. 1780. pages 63.

30. *Tho. Hoffmann*, M. D. de præsagiis tempestatis naturalibus, Dissertatio Medico-physica. Editio secunda. 8vo. Basil, 1781. p. 48.

31. *Sebaldi Justini Brugmans* Lithologia Groningana juxta ordinem Wallerii digesta. Cum synonymis aliorum imprimis Linnæi et Cronstedii cum figuris æneis. Publicé defensa cum summos in Philosophia honores in academia Groningo-omlandica consequeretur. 8vo. Groningæ, 1781. 120 pages.

32. Differtatio Inauguralis de Gardenia, quam prefide *C. P. Thunberg* fuftinuit *Petrus Djupedius.* 4to. Upfal, 1781.

Only one fpecies of this plant, which is remarkable for the beauty of its flowers, has been hitherto known. Dr. Thunberg here defcribes eight fpecies, of five of which he gives engravings.

33. Adverfaria Medica, pars prima, fpectat ad morbos pectoris inflammatorios fpeciatim pleuritidem tum fanguineam tum biliofam. Auctore D. *Angel. Galli.* 8vo. Pavia, 1781.

34. Differtatio Medica de ufu cantharidum interno. Auctore *Johanne Conrad Stockar von Neuforn.* 4to. Gottingæ, 1781.

35. Progymnafma Medico-practicum de Metaftafi lactis. Auctore *Petro Sylveftre.* 4to. Montpellier, 1781. p. 47.

36. Occurfus Medici de Vaga ægritudine infirmitatis nervorum *Andreæ Comparetti.* 8vo. Venetiis, 1780. p. 396.

37. Dictionnaire de Santé. *i. e.* a Dictionary of health. Fifth edition. 8vo. Paris, 1783. 3 vols.

